# Obstructive sleep apnea: a major risk factor for COVID-19 encephalopathy?

**DOI:** 10.1186/s12883-023-03393-2

**Published:** 2023-09-27

**Authors:** Gautier Breville, François Herrmann, Dan Adler, Christine Deffert, Giulia Bommarito, Patrick Stancu, Alice Accorroni, Marjolaine Uginet, Frederic Assal, Renaud Tamisier, Patrice H. Lalive, Jean-Louis Pepin, Karl-Olof Lövblad, Gilles Allali

**Affiliations:** 1 Department of Neurology, Perelman School of Medicine, University of Pennsylvania, Philadelphia, USA; 2 Department of Neurosciences, Division of Neurology, Geneva University Hospitals, Geneva, Switzerland; 3 Department of Rehabilitation and Geriatrics, Division of Geriatrics, Geneva University Hospitals and University of Geneva, Geneva, Switzerland; 4 Division of Pneumology, La Tour Hospital, Geneva, Switzerland; 5 Laboratory of Biological Fluids, Laboratory Medicine Division, Diagnostic Department, Geneva University Hospitals, Geneva, Switzerland; 6 Laboratory Medicine Division, Department of Medical Specialties, Faculty of Medicine, University of Geneva, Geneva, Switzerland; 7 Leenaards Memory Center, Department of Clinical Neurosciences, Lausanne University Hospital and University of Lausanne, Lausanne, Switzerland; 8 Univ. Grenoble Alpes, INSERM, CHU Grenoble Alpes, HP2, 38000 Grenoble, France; 9 Department of Pathology and Immunology, Faculty of Medicine, University of Geneva, Geneva, Switzerland; 10 Diagnostic Department, Division of Laboratory Medicine, Geneva University Hospitals, Geneva, Switzerland; 11 Division of Neuroradiology, Geneva University Hospitals and University of Geneva, Geneva, Switzerland; 12 Department of Neurology, Division of Cognitive and Motor Aging, Albert Einstein College of Medicine, Yeshiva University, Bronx, NY USA

**Keywords:** SARS-CoV-2, COVID-19 encephalopathy, Obstructive sleep apnea

## Abstract

**Background:**

This study evaluates the impact of high risk of obstructive sleep apnea (OSA) on coronavirus disease 2019 (COVID-19) acute encephalopathy (AE).

**Methods:**

Between 3/1/2020 and 11/1/2021, 97 consecutive patients were evaluated at the Geneva University Hospitals with a neurological diagnosis of COVID-19 AE. They were divided in two groups depending on the presence or absence of high risk for OSA based on the modified NOSAS score (mNOSAS, respectively ≥ 8 and < 8). We compared patients’ characteristics (clinical, biological, brain MRI, EEG, pulmonary CT). The severity of COVID-19 AE relied on the RASS and CAM scores.

**Results:**

Most COVID-19 AE patients presented with a high mNOSAS, suggesting high risk of OSA (> 80%). Patients with a high mNOSAS had a more severe form of COVID-19 AE (84.8% versus 27.8%), longer mean duration of COVID-19 AE (27.9 versus 16.9 days), higher mRS at discharge (≥ 3 in 58.2% versus 16.7%), and increased prevalence of brain vessels enhancement (98.1% versus 20.0%). High risk of OSA was associated with a 14 fold increased risk of developing a severe COVID-19 AE (OR = 14.52).

**Discussion:**

These observations suggest an association between high risk of OSA and COVID-19 AE severity. High risk of OSA could be a predisposing factor leading to severe COVID-19 AE and consecutive long-term sequalae.

**Supplementary Information:**

The online version contains supplementary material available at 10.1186/s12883-023-03393-2.

## Introduction

Coronavirus disease 2019 (COVID-19) causes extrapulmonary manifestations, [1] including acute encephalopathy (AE) [2–4]. The COVID-19 AE has various clinical expressions ranging from subtle cognitive disturbances (subsyndromal delirium) to coma. Its physiopathology is not fully understood and goes beyond a metabolic disorder such as electrolytic imbalance, renal or hepatic failure, persistence of sedative effects or hypoxemia that are lacking in many COVID-19 AE patients.

The spike protein of the severe acute respiratory syndrome coronavirus 2 (SARS-CoV-2) binds its cellular receptor, the angiotensin-converting enzyme 2 (ACE2), expressed in nasal and bronchial epithelial cells, pneumocytes and brain vascular endothelium.[5] Endotheliopathy has been recognized as one of the main pathogenic mechanism in COVID-19, including cerebral arteries involvement [3, 6], that may be responsive to high-dose glucocorticoids [7]. In a cohort of 31 patients suffering from COVID-19 AE at the Geneva University Hospitals (Geneva, Switzerland), we observed an increased prevalence of gadolinium enhancement in large arteries on brain magnetic resonance imaging (MRI) (90.6%) suggestive of an underlying cerebral endotheliitis [8].

Patients with obstructive sleep apnea (OSA) are reported to be at increased risk for COVID-19 infection [9–11]. OSA-induced intermittent hypoxia triggers a pro-inflammatory state, that promote the development of endothelial dysfunction, [12, 13] and this dysfunction has been reported to be improved with OSA therapy [14, 15]. The diagnosis of definite OSA requires overnight polygraphy or polysomnography (gold standard) [16]. The burden of the disease affects nearly one billion people worldwide [16] and limited accessibility and availability of these investigations explain why OSA is widely underdiagnosed [17, 18]. The development and validation of the NOSAS score has enabled to classify patients at high risk for OSA with the five following items: neck circumference, obesity, snoring, age and sex. Using a threshold of ≥ 8 points, NOSAS identifies individuals at-risk for significant OSA with an area under the curve of 074 (0·72–0·76) [19].

As OSA and severe COVID-19 might exert synergistic effects for endothelial injury, we investigated a cohort of 97 patients hospitalized for COVID-19 AE and computed a modified NOSAS score (mNOSAS). Therefore, we framed the study with the following aims: (i) primary outcome focused on the association between OSA as assessed by the mNOSAS score and the severity of COVID-19 AE. Secondary outcomes evaluated (ii) the duration of COVID-19 AE, (iii) the disability at discharge (modified Ranking Scale) as well as (iv) the impact of associated OSA on gadolinium enhancement in large arteries on brain MRI. Based on the position paper recently published, we also looked at (v) inflammatory biomarkers both in the blood and the cerebrospinal fluid (CSF) [20].

## Materials and methods

### Study population

**Fig. 1:**
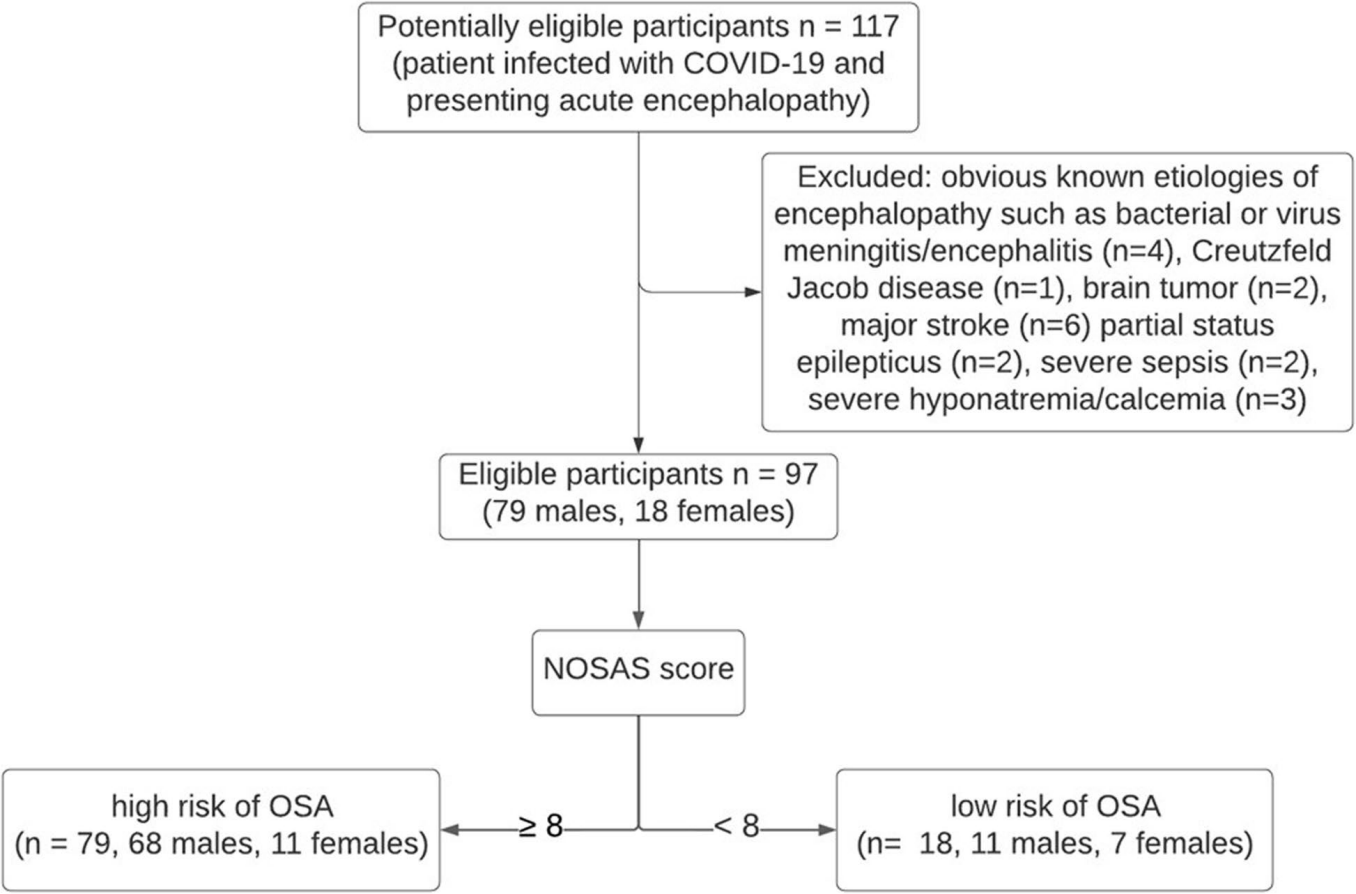
Flow chart

Between March 2020 and November 2021, 117 COVID-19 patients referred to the Geneva University Hospitals were evaluated by a board-certified neurologist with a diagnosis of AE. SARS-CoV-2 infection was documented by a positive SARS-CoV-2 reverse transcription‐polymerase chain reaction (RT‐PCR) assay from a nasopharyngeal swab at the time of the hospitalization. Inclusion criteria for COVID-19 AE were defined by a rapidly developing (less than 4 weeks) pathobiological process in the brain leading to delirium, decreased level of consciousness or coma [21]. Here, we focused on a series of patients with delirium or subsyndromal delirium (according to the definition of the consensus statement) [21] at the time of the neurological assessment. Exclusion criteria were AE related to electrolyte disturbances, infection, drug or alcohol toxicity and/or withdrawal, metabolic disorders, low perfusion state or acute central nervous system conditions, such as major stroke, brain tumor, encephalitis, meningitis or Creutzfeldt Jacob disease (*n* = 20) (Fig. 1). Patients with minor stroke were included in our cohort. The severity of the COVID-19 AE was based on the Richmond Agitation Sedation Scale (RASS) and the Confusion Assessment Method (CAM): severe cases were defined on a RASS <  − 3 at worst presentation ─ meaning deep sedation, no response to voice but possible movement or eye, opening to physical stimulation; or on a CAM score ≥ 3 among patients with a RASS ≥ -3 ─ meaning displaying 3 out of 4 items among symptoms fluctuation, inattention, thought disturbance, and altered alertness.

The NOSAS score classifies patients at high risk for significant OSA with the following items: neck circumference, obesity, snoring, age and sex (NOSAS). The NOSAS score allocates 4 points for neck circumference > 40 cm; 3 points for body mass index (BMI) ≥ 25 and < 30 kg/m^2^; 5 points for BMI ≥ 30 kg/m^2^ (obesity); 2 points for snoring; 4 points if Age > 55 years and 2 points for men (sex). Neck circumference was measured between mid-cervical spine and mid-anterior neck [19]. Overweight was defined as a body mass index (BMI) > 25 kg/m^2^. Snoring information was recorded for only 19 patients (missing data: 78), so we used a mNOSAS score that did not include snoring information. Among patients presenting with COVID-19 AE, we divided them into two groups according to the mNOSAS score: one group called “high risk of OSA group” with a mNOSAS score ≥ 8 gathering patients with high probability of OSA (*n* = 79) and one group called “no OSA” group with a mNOSAS score < 8 signifying low probability of OSA (*n* = 18) [19]. Some of the patients have had polysomnography with final diagnosis of definite OSA (gold standard). Among them, the apnea hypopnea index ─ the number of apneas or hypopneas recorded per hour of sleep ─ was used to indicate the severity of definite OSA. Based on the apnea hypopnea index, the severity of OSA could be classified as follows: None/Minimal: < 5 per hour; Mild: ≥ 5, but < 15 per hour; Moderate: ≥ 15, but < 30 per hour; Severe: ≥ 30 per hour [12, 16]. Among patients with definite OSA, some of them usually use night devises such as continuous positive airway pressure (CPAP) or oral appliances like the mandibular advancement device [12].

The mRS is widely used to assess global outcome after stroke [22]. We used the mRS at discharge to estimate the disability of COVID-19 AE patients at discharge.

Preexisting cognitive disorder was defined as any cognitive disorder impairment by a neurologist before the beginning of COVID-19 acute encephalopathy. Preexisting heart and respiratory disease were defined as any heart or respiratory disease diagnosed by a cardiologist, a pulmonologist or a general practitioner prior to the onset of COVID-19 acute encephalopathy. Toxic use was reported by the physician in charge at the time of hospital admission (anamnesis/heteroanamnesis).

The study was approved by the institutional review board of the Geneva University Hospitals (protocol #2020‐01206—approved May 25, 2020).

### Paraclinical examinations: CT scan, MRI, EEG, blood and CSF tests

Electronic medical records, pulmonary CT scan, brain MRI, electroencephalogram (EEG), blood, and CSF samples were based upon prospective descriptive assessment of the patients during their hospitalization. All clinical and neurological manifestations of each patients were evaluated by at least one trained neurologist resident and one senior neurologist.

Pulmonary CT scan were performed to assess the percentage of lung parenchyma with COVID-19 lesions: mild (0–30%), moderate (30–50%), severe (50–70%), very severe (> 70%).

The brain MR images were acquired on a 1.5 T clinical scanner (Philips Ingenia (Philips Medical Systems, Eindhoven, The Netherlands)) equipped with a head and neck coil. The protocol included an axial T1 and T2-weighted, axial diffusion-weighted imaging (DWI), susceptibility-weighted images (SWI) for the detection of blood, as well as 3D time-of-flight (TOF) MR angiography (MRA) of the intracranial vessels, and a dynamic 3D contrast-enhanced MRA of the neck vessels from the aortic arch to the circle of Willis. Precontrast and postcontrast fat-saturated T1-weighted black blood VISTA images in all patients (TE: 17 ms, TR: 400 ms, image thickness 1.5 mm) were acquired in the axial and coronal planes. Measurement of the longitudinal length of the arterial wall enhancement was manually performed on the injected T1 weighted MR images. All MRI were blindly reviewed by two board-certified neuroradiologists and contrast vessel enhancement was validated when common agreements were reached. Additionally, they looked for the presence of hypointensities on SWI as signs of microbleeds, and for ischemia on the diffusion weighed MR images. The MR angiographic images were evaluated for the presence of arteriosclerotic changes. During contrast administration, a 3D angiogram (TE: 1.98 ms, TR: 5.6 ms, 1.10‐mm thick slices) of the carotids was additionally performed as well as post‐contrast T1 axial images (TE, 2.46 ms; TR, 262 ms; 5‐mm thick slices) over the brain. Inflammation of vessel walls was suspected when contrast enhancement of the vessel wall was homogeneous, and we defined circumferential inflammation of vessel wall when contrast enhancement was greater than 50% of the circumference [23, 24]. Inflammatory atheromatous plaques, as a potential cause of such intracranial vessel enhancement, were excluded by angio‐MR, angio‐CT or echo‐doppler.

A standard video-EEG in accordance with the international 10–20 system was recorded in 80 patients (82.5%) ─ 67 patients (69.1%) included in the OSA group and 13 (72.2%) in the no OSA group.

All patient underwent blood tests including C-reactive protein (CRP) and leucocyte, lymphocyte, segmented neutrophil, monocyte and thrombocyte counts. CSF spinal taps were performed in 37 patients (38.1%) — 33 (41.8%) in the OSA group and 4 (22.2%) in the no OSA group (Supplemental Table 3).

### Statistical analysis

Baseline characteristics were summarized using means and standard deviations (SD) or median and interquartile range (IQR) or frequencies and percentages, as appropriate. Between groups comparisons (high risk for OSA versus not at high risk for OSA) were performed using unpaired t test, Mann Whitney u test, or Fisher exact test, as appropriate. We performed stepwise forward multiple logistic regression models to identify which combination of symptoms or paraclinical parameter was associated with COVID-19 AE among the two groups. The proportion of the variance explained by the models was estimated by the pseudo-R^2^. All statistical analyses were performed using STATA software version 17.0.

## Results

Demographic and clinical characteristics between high risk of OSA and no OSA patients were reported in Table 1. The mean age of patients was 69.39 ± 10.13 years with an expected male predominance in the OSA group (86.1% versus 61.1%, *p* = 0.038). All 27 patients (27.8% of the cohort) with definite OSA based on polysomnography had a mNOSAS score ≥ 8 and so belonged to the OSA group (34.2% of the OSA group).

**Table 1:** Demographic characteristics

variables	Total (*n* = 97)	High risk of OSA (*n* = 79, 81.4%)	no OSA (*n* = 18, 18.6%)	*p*-value
age (at admission)	69.4 (± 10.1)	70.3 (± 9.7)	65.3 (± 11.3)	0.096^2^
male	79 (81.4%)	68 (86.1%)	11 (61.1%)	0.038^1^
education degree^a^				0.774^1^
1	14 (17.9%)	11 (16.9%)	3 (23.1%)	
2	36 (46.2%)	31 (47.7%)	5 (38.5%)	
3	28 (35.9%)	23 (35.4%)	5 (38.5%)	
≥ 1 vascular risk factors	74 (76.3%)	63 (79.7%)	11 (61.1%)	0.124^1^
Body Mass Index (BMI)	27.97 (± 5.57)	28.99 (± 5.62)	23.5 (± 2.22)	< 0.001^2^
smoking	16 (16.8%)	14 (17.9%)	2 (11.8%)	0.728^1^
blood pressure hypertension	63 (64.9%)	53 (67.1%)	10 (55.6%)	0.416^1^
diabete	36 (37.1%)	31 (39.2%)	5 (27.8%)	0.427^1^
dyslipidemia	34 (35.1%)	29 (36.7%)	5 (27.8%)	0.589^1^
Definite OSA^b^	27 (27.8%)	27 (34.2%)	0	0.003^1^
apnea hypopnea index (/h)^b^	53.68 (± 24.92)	53.68 (± 24.92)	-	-
night breath device for OSA^b^	13 (13.4%)	13 (16.5%)	0	-
modified NOSAS score^b^	11.12 (± 3.62)	12.49 (± 2.28)	5.11 (± 1.81)	< 0.001^2^
preexisting cognitive disorder^c^	17 (17.7%)	14 (17.9%)	3 (16.7%)	0.999^1^
preexisting heart disease^c^	18 (18.6%)	16 (30.2%)	2 (18.2%)	0.714^1^
preexisting respiratory disease^c^	9 (9.3%)	8 (15.1%)	1 (9.1%)	0.999^1^
toxic use^c^	10 (10.3%)	9 (11.4%)	1 (5.6%)	0.683^1^

*Abbreviation*: *OSA* Obstructive sleep apnea

^1^Fisher’s exact test. Table results were given in number of patients (percentage of total number of patients per group)

^2^t-test. Table results were given in median (± interquartile ratio)

^a^Education degree was defined as followed: 1 = primary education, 2 = lower secondary education, 3 = upper secondary education

^b^Definite OSA was assessed by polysomnography (gold standard) [12, 15]. The Apnea Hypopnea Index was used to indicate the severity of definite OSA. The Apnea Hypopnea Index is the number of apneas or hypopneas recorded per hour of sleep (number of events per hour). Based on the Apnea Hypopnea Index, the severity of OSA is classified as follows: None/Minimal: < 5 per hour; Mild: ≥ 5, but < 15 per hour; Moderate: ≥ 15, but < 30 per hour; Severe: ≥ 30 per hour [12, 15]. Among patients with definite OSA, some of them usually used night devises such as continuous positive airway pressure (CPAP) or oral appliances like the mandibular advancement device [12]. The NOSAS score classified patients at high risk for significant OSA with the following items: neck circumference, obesity, snoring, age and sex (NOSAS) [18]. Snoring information was recorded for only 19 patients (missing data: 78), so we used a modified NOSAS score that did not include snoring information

^c^Preexisting cognitive disorder was defined as any cognitive disorder impairment by a neurologist before the beginning of COVID-19 acute encephalopathy. Preexisting heart and respiratory disease were defined as any heart or respiratory disease diagnosed by a cardiologist, a pulmonologist or a general practitioner prior to the onset of COVID-19 acute encephalopathy. Toxic use was reported by the physician in charge at the time of hospital admission (anamnesis/heteroanamnesis)

Table 2 shows patients’ characteristics at the time of COVID-19 onset (general examination data and COVID-19 pulmonary imaging status) and at COVID-19 AE paroxysm (neurological signs, encephalopathy features, epidemiological features, biological data from blood and CSF, brain MRI data and EEG results). At the time of COVID-19 onset, common general symptoms were dyspnea (55.7%), cough (63.2%) and fever (82.5%). Oxygen needs at admission was FiO2 32.31% (± 17.30), 27.84% (± 11.38) in the no OSA group compared to 33.35% (± 18.31) in the OSA group (*p* = 0.113). The percentage of COVID-19 related pulmonary lesions determined with pulmonary CT scan was similar between the two groups (*p* = 0.963).

**Table 2:** Patient characteristics at the time of COVID-19 onset and COVID-19 acute encephalopathy

variables	Total (*n* = 97)	High risk of OSA (*n* = 79, 81.4%)	no OSA (*n* = 18, 18.6%)	*p*-value
**General examination and parameter at COVID-19 onset**				
dyspnea	54 (55.7%)	42 (53.2%)	12 (66.7%)	0.431^1^
cough	60 (63.2%)	50 (64.9%)	10 (55.6%)	0.588^1^
fever	80 (82.5%)	67 (84.8%)	13 (72.2%)	0.299^1^
FiO2	32.31 (± 17.30)	33.35 (± 18.31)	27.84 (± 11.38)	0.113^2^
**Percentage of lung parenchyma with COVID-19 lesions (pulmonary CT scan)**				0.583^2^
0–30%	13 (20.0%)	11 (20.4%)	2 (18.2%)	
30–50%	22 (33.8%)	19 (35.2%)	3 (27.3%)	
50–70%	17 (26.2%)	14 (25.9%)	3 (27.3%)	
> 70%	13 (20.0%)	10 (18.5%)	3 (27.3%)	
**Neurological signs at COVID-19 acute encephalopathy**				
fluctuation	87 (89.7%)	73 (92.4%)	14 (77.8%)	0.085^1^
inattention	86 (88.7%)	74 (93.7%)	12 (66.7%)	0.005^1^
thought disturbance	68 (74.7%)	61 (82.4%)	7 (41.2%)	0.001^1^
alertness trouble	48 (48.5%)	44 (54.4%)	4 (22.2%)	0.026^1^
drowsiness	51 (52.6%)	42 (53.2%)	9 (50.0%)	0.999^1^
agitation	33 (34.0%)	29 (36.7%)	4 (22.2%)	0.283^1^
psychomotor slowdown	63 (65.6%)	55 (70.5%)	8 (44.4%)	0.053^1^
obnubilation	32 (34.4%)	28 (36.8%)	4 (23.5%)	0.401^1^
perseveration	53 (58.2%)	47 (64.4%)	6 (33.3%)	0.031^1^
disorientation	48 (54.5%)	43 (60.6%)	5 (29.4%)	0.030^1^
hallucination	17 (19.3%)	14 (19.4%)	3 (18.8%)	0.999^1^
focal neurological sign	27 (27.8%)	23 (29.1%)	4 (22.2%)	0.772^1^
**COVID-19 acute encephalopathy features**				
CAM	2.97 (± 1.05)	3.18 (± 0.89)	2.06 (± 1.21)	0.001^3^
RASS ≤ -3	8 (8.2%)	7 (8.9%)	1 (5.6%)	0.168^1^
mutism	14 (14.4%)	12 (15.2%)	2 (11.1%)	0.999^1^
severe encephalopathy^a^	72 (74.2%)	67 (84.8%)	5 (27.8%)	< 0.001^1^
duration of encephalopathy (days)	25.8 (± 59.3)	27.9 (± 65.5)	16.9 (± 12.4)	0.018^3^
**Biological results in the blood**				
C-reactive protein (mg/l)	89.85 (39.2–163.4)	89.6 (41.9—163.6)	90.1 (9.7—159.3)	0,504^2^
leucocytes (/mm3)	9.88 (7.3–12.2)	9.84 (7.2—12)	11.35 (8.5—14.2)	0,215^2^
lymphocytes (/mm3)	0.82 (0.5–1.1)	0.79 (0.5—1)	0.97 (0.7—1.2)	0,230^2^
segmented neutrophils (/mm3)	7.47 (5.5–10.4)	7.35 (5.1—10)	9.09 (6.4—11.7)	0,272^2^
monocytes (/mm3)	0.53 (0.3–0.8)	0.52 (0.3—0.8)	0.58 (0.4—0.7)	0,998^2^
thrombocytes (/mm3)	298 (232–354.5)	307 (241—356.8)	264 (167—319)	0,345^2^
**Brain MRI**				
*missing values*^b^	35	27	8	
leucoencephalopathy				0.050^1^
0	23 (31.5%)	17 (28.3%)	6 (46.2%)	
1	32 (43.8%)	30 (50.0%)	2 (15.4%)	
2	7 (9.6%)	6 (10.0%)	1 (7.7%)	
3	11 (15.1%)	7 (11.7%)	4 (30.8%)	
stroke (DWI lesion)	34 (47.2%)	26 (44.1%)	8 (61.5%)	0.359^1^
hyperT2 lesion (number)	7.12 (± 6.22%)	7.3 (± 6.2)	6.31 (± 6.54)	0.623^3^
microbleed (number)	3.03 (± 7.43%)	2.9 (± 7.75)	3.62 (± 5.92)	0.714^3^
number of vessels with endotheliitis^c^				< 0.001^1^
0	9 (14.5%)	1 (1.9%)	8 (80.0%)	
1	18 (29.0%)	16 (30.8%)	2 (20.0%)	
2	16 (25.8%)	16 (30.8%)	0	
3	19 (30.6%)	19 (36.5%)	0	
circumferential endotheliitis^c^	48 (77.4%)	48 (92.3%)	0	< 0.001^1^
endotheliitis^c^				< 0.001^1^
unilateral	26 (41.9%)	24 (46.2%)	2 (20.0%)	
bilateral	27 (43.5%)	27 (51.9%)	0	
stenosis	3 (4.1%)	3 (5.0%)	0	
**EEG slowing**	47 (53.4%)	40 (55.6%)	7 (43.8%)	0.420^1^
**Epidemiological features**				
intensive care unit	62 (63.9%)	50 (63.3%)	12 (66.7%)	0.999^3^
hospitalization time	43.5 [25.0–61.5]	43.5 [25.0–61.0]	42.5 [28.0–66.0]	0.796^2^
mRS at discharge				0.008^1^
0	9 (9.3%)	8 (10.1%)	1 (5.6%)	
1	23 (23.7%)	13 (16.5%)	10 (55.6%)	
2	16 (16.5%)	12 (15.2%)	4 (22.2%)	
3	22 (22.7%)	21 (26.6%)	1 (5.6%)	
4	14 (14.4%)	13 (16.5%)	1 (5.6%)	
5	1 (1.0%)	1 (1.3%)	0 (0.0%)	
6 (death)	12 (12.4%)	11 (13.9%)	1 (5.6%)	
mRS at discharge ≥ 3	49 (50.5%)	46 (58.2%)	3 (16.7%)	0.002^1^

This table presents patient characteristics at the time of COVID-19 onset (general examination data and COVID-19 pulmonary imaging status) and at COVID-19 acute encephalopathy paroxysm (neurological signs, encephalopathy features, epidemiological features, biological data from blood and CSF, brain MRI data and EEG results)

*Abbreviations*: *CAM* Confusion Assessment Method, *COVID-19 AE* COVID-19 acute encephalopathy, *DWI* Diffusion-weighted imaging, *OSA* Obstructive sleep apnea, *mRS* modified Rankin Scale, *RASS* Richmond Agitation Sedation Scale

^1^Fisher's exact test. Table results were given in number of patients (percentage of total number of patients per group)

^2^Mann-Whitney u test. Table results were given in median (± interquartile ratio)

^3^t-test. Table results were given in median (± interquartile ratio)

^a^severe encephalopathy was defined on a RASS <  − 3 at worst presentation ─ meaning deep sedation, no response to voice but possible movement or eye, opening to physical stimulation; or on a CAM score ≥ 3 among patients with a RASS ≥ -3 ─ meaning displaying 3 out of 4 items among symptoms fluctuation, inattention, thought disturbance, and altered alertness

^b^Missing value. Brain MRI were missing in many patients because of the inability to perform these tests due to patient compliance at the acute phase of COVID-19 encephalopathy

^c^The term “endotheliitis” referred to homogeneous gadolinium contrast enhancement of the inner part of the vessel wall (injected brain MRI) without stenosis. Circumferential endotheliitis referred to contrast enhancement of the vessel wall greater than 50% of the circumference

Neurological signs at COVID-19 AE paroxysm were also presented in Table 2. Patients with high risk of OSA versus no OSA presented more often with inattention (93.7% vs. 66.7%; *p* = 0.005), thought disturbance (82.4% vs. 41.2%; *p* = 0.001), alertness trouble (54.4% vs. 22.2%, *p* = 0.026), perseveration (64.4% vs. 33.3%; *p* = 0.031), and disorientation (60.6% vs. 29.4%; *p* = 0.030). Patients with high risk of OSA exhibited more often severe COVID-19 AE on neurological examination compared to those without OSA (84.8% vs. 27.8%; *p* < 0.001). The mean CAM score was significantly different in the two groups: 3.18 (standard deviation (SD) ± 0.89) in the high risk of OSA group versus 2.06 (± 1.21) in the no OSA group (*p* = 0.001). The mean duration of COVID-19 AE significantly differed between the two groups: 27.9 (± 65.5) days in the high risk of OSA group versus 16.9 (± 12.4) in the no OSA group (*p* = 0.018).

The two groups did not significantly differ in blood biological data (Table 2). Notably, the mean serum concentration of C‐reactive protein was 89.85 (39.2–163.7) mg/L. Only 37 patients (38.1%) had a CSF analysis (Supplemental Table 3). Of these, CSF white blood cell count was normal in 35 patients (94.6%): all patients in the no OSA group had white blood cell count ≤ 1 /cm^3^ while 18 (54%) patients of the high risk of OSA group have white blood cell count ≥ 2 /cm^3^. The mean CSF white blood cell count was 4.3 (± 15.5) /cm^3^ in the high risk of OSA group compared to 0.5 (± 0.6) /cm^3^ in the no OSA group (*p* = 0.056). The mean CSF lymphocyte count was 2.1 (± 2.0) /cm^3^ in the high risk of OSA group compared to 0.9 (± 0.1) /cm^3^ in the no OSA group (*p* = 0.005). The mean CSF macrophages count was 1.1 (± 1.3) (%) in the high risk of OSA group compared to 0.4 (± 0.5) (%) in the no OSA group (*p* = 0.301), but 7 patients (21%) of the high risk of OSA group have more than 1% of activated macrophages compared to none in the no OSA group. The rest of CSF analyses did not evidence any difference between the 2 groups. RT-PCR for SARS-CoV-2 was negative for all patients in the CSF.

**Fig. 2:**
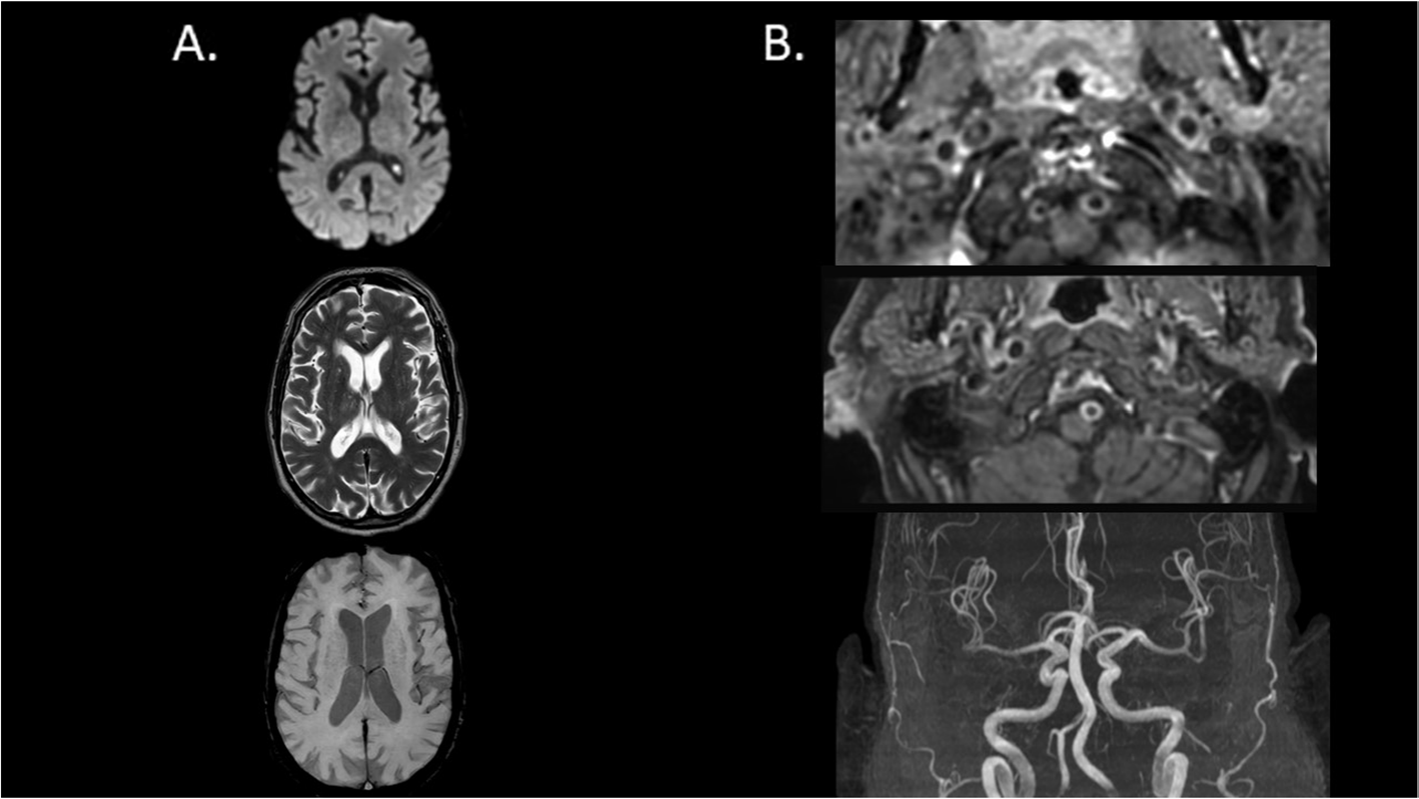
Brain MRI of one patient from the OSA group with evidence of intracranial vessel gadolinium enhancement without vascular stenosis nor any parenchymal lesion. Legend: **A** from the top to the bottom, we show brain axial slides of diffusion-weighted imaging (DWI, top), T2-weighted (middle) and susceptibility-weighted images (SWI, bottom) brain MRI sequences. They do not find any brain parenchymal damage. **B** from the top to the bottom, we show axial slide of dynamic 3D contrast-enhanced MR angiography (MRA) of the neck vessels at the vertebral arteries levels (top) and at the basilar artery level (middle) as well as 3D time-of-flight (TOF) MRA of the intracranial vessels (bottom). The first two images show gadolinium contrast enhancement of both vertebral arteries and basilar artery, without any vascular stenosis (TOF, third image)

Injected MRI was available for 62 patients (63.9%) — 52 (65.8%) in the high risk of OSA group and 10 (55.6%) in the no OSA group (Table 2). Noteworthy, intracranial vessel gadolinium enhancement was observed in 53 patients (85.5%), with predominance in the high risk of OSA group (98.1%) compared to no OSA group (20%) (*p* < 0,001). The vast majority of the vessel enhancement was circumferential (48 patients, 77.4%) and found on vertebral arteries without sign of stenosis or downstream ischemia. Among patients with intracranial vessel gadolinium enhancement, the number of enhancing vessels was significantly higher in the high risk of OSA group compared to the no OSA group ─ respectively 35 patients (67.3%) in the high risk of OSA group versus none in the no OSA group with more than one enhancing vessel (*p* < 0,001). Bilateral intracranial enhancing vessel involvement was only described in the high risk of OSA group ─ 27 patients (51.9%, *p* < 0,001). Cerebral microbleeds were reported in 56 of 97 patients (57.7%). The mean number of microbleeds by patients was 3.03 (± 7.43) with no difference in the two groups. Finally, there were no differences in term of brain MRI parenchyma abnormalities (acute stroke, T2-FLAIR hyperintensities, microbleeds) between the two groups. The Fig. 2 illustrates MRI sequences of one patient from the high risk of OSA group with evidence of intracranial vessel gadolinium enhancement without vascular stenosis nor any parenchymal lesion.

No ictal discharge was reported at electroencephalogram (EEG), while EEG slowing was noticed in 47 of 97 patients (53.4%) with no difference between the groups.

The modified Rankin scale (mRS) at discharge was statistically significantly different between the two groups (*p* = 0.008). Forty-six patients (58.2%) from the high risk of OSA group presented mRS ≥ 3 meaning moderate to severe deficit or death, as compared to three patients (16.7%) in the no OSA group (*p* = 0.002). Twelve patients included in the current series died during their hospitalization, 11 in the high risk of OSA group (13.9%) and 1 in the no OSA group (5.6%, *p* = 0.45). The median length of hospital stay was similar between the two groups (44 (27–61.8) days) without any difference between the two groups: 44.5 (26.5–61.2) in the high risk of OSA group compared to 42.5 (30–62.8) in the no OSA group (*p* = 0.594).

Using a stepwise forward multiple logistic regression model, high risk for significant OSA based on the mNOSAS score was selected by the model with a 14 times risk of developing a severe COVID-19 AE (OR = 14.52; 95% CI [4.37–48.22]; *p* < 0.001), explaining 19.9% of the variability of the severity. Using the same method, the age at admission (OR = 1.07; 95% CI [1.01–1.13]; *p* = 0.018), the mNOSAS score (OR = 1.17; 95% CI [0.99–1.38]; *p* = 0.058), reduced dyspnea sensation at admission (OR = 0.28; 95% CI [0.10–0.79]; *p* = 0.017) and the severity of COVID-19 AE (OR = 7.37; 95% CI [1.87–29.02]; *p* = 0.004) were selected by the model to be associated with the risk of developing severe disability at discharge (mRS score ≥ 3), explaining 29.1% of the variability of the disability at discharge (see Table 3).

**Table 3:** Multiple logistic regression analyses showing that high risk for significant OSA based on the mNOSAS score was selected by the model with a 14 times risk of developing a severe COVID-19 AE (A), and that the age at admission, the mNOSAS score, reduced dyspnea sensation at admission and the severity of COVID-19 AE were associated with the risk of developing severe disability at discharge (mRS score ≥ 3)

**A**				
**Severe COVID-19 AE**	**Odds ratio**	***p* value**	**[95% Confident Interval]**	
Modified NOSAS score	14.52	< 0.001	4.37	48.22
**B**				
**mRS**	**Odds ratio**	***p*** **value**	**[95% Confident Interval]**	
Age at admission	1.07	0.018	1.01	1.13
Modified NOSAS score	1.17	0.058	0.99	1.38
Reduced dyspnea at admission	0.28	0.017	0.10	0.79
Severe COVID-19 AE	7.37	0.004	1.87	29.02

*Abbreviations*: *COVID-19 AE* COVID-19 acute encephalopathy, *OSA* Obstructive sleep apnea, *mRS* modified Rankin Scale

We conducted a sensitivity analysis in which we compared the no OSA group (*n* = 18) with the group confirmed to have definitive OSA through polysomnography, termed the OSA group (*n* = 27). The results confirmed the findings of the study (see Supplemental Tables 1 and 2).

## Discussion

In this cohort of patients with COVID-19 AE, most patients were at high risk for OSA as assessed by the mNOSAS score ≥ 8 (> 80% of COVID-19 AE patients). Patients with high risk of OSA were more prevalent in our COVID-19 AE population compared to the global burden of disease in the general population in Switzerland [16]. Among patients with COVID-19 AE, those with a high risk of OSA are at risk for (i) severe COVID-19 AE, (ii) longer duration of COVID-19 AE, (iii) a higher mRS at discharge suggestive of long-term disability, and (iv) a gadolinium vessel enhancement of brain arteries evoking an inflammation of the vessel endothelia or “endotheliitis” at the acute phase. This association between high risk of OSA and COVID-19 AE severity and related-disability represents the main study findings and confirmed our previous hypothesis [20].

High risk of OSA increases by 14 times the risk of developing a severe COVID-19 AE, explaining around 20% of the variability of COVID-19 AE severity. Consistently, a previous cohort of 140 COVID-19 patients hospitalized in the Intensive Care Unit found similar results with 118 patients (84.3%) presenting with delirium or an abnormal neurological examination. Fifteen (12.7%) had a pre-existing OSA in the delirium or abnormal neurological examination group, whereas only one of 22 patients (4.5%) had a pre-existing OSA in the no-delirium and normal neurological examination group [4].

Moreover, the association of the age at admission, the mNOSAS score, the low dyspnea sensation at admission and the severity of COVID-19 AE are associated with the risk of developing disability at discharge (mRS score ≥ 3). Of note, the absence of breathlessness sensation is strongly associated with poor COVID-19 disability outcome [25]. This observation supports the hypothesis that COVID-19 encephalopathic patients may experiment “silent” or “happy” hypoxemia: i.e. the lack of a subjective experience of breathing discomfort [25, 26], ─ despite profound hypoxemia and altered lung mechanics at the initial stages of SARS-CoV2 infection. This lack of breathlessness might represent another COVID-19 neurological consequence.

The high rate of endotheliitis at brain imaging among encephalopathic patients with high risk of OSA supports the hypothesis of an endothelial vulnerability in the OSA population that could be a major predisposing factor leading to severe COVID-19 AE and long-term sequalae [20]. Although the number of performed lumbar puncture (LP) is insufficient to bring strong conclusion on CSF analyses (inability to perform LP due to patient compliance at the acute phase), elevated CSF lymphocytes and the trend toward elevated CSF activated macrophages in COVID-19 AE patients with high risk of OSA may suggest a basal pro-inflammatory state that could be enhanced during SARS-CoV2 infection. OSA-induced intermittent hypoxia is known to lead to a pro-inflammatory immunological state [12, 13]. A meta-analysis demonstrated that OSA is independently associated with an increased risk of endothelial dysfunction proportionally to the severity of intermittent hypoxia [12]. Untreated OSA patients display higher levels endothelial cell oxidative stress, circulating endothelin and a pro-inflammatory state, facilitating endothelial injury and dysfunction, and preventing appropriate repair endothelium capacity [12, 13]. OSA is thus responsible for blood vessel walls remodeling leading to increased permeability due to endothelial cell disruption and impaired recycling [27, 28]. Serum from untreated OSA patients alters in vitro endothelial cell repair function and activates monocyte migration. This may be related to an unfavorable balance between the pro healing (VEGF) and pro injury (CRP) factors that may promote vascular injury in OSA [29]. These pathophysiological explanations support our observations that patients with OSA may have an endothelial vulnerability that puts them at higher risk of developing severe complications related to SARS-CoV-2 inflammation, including COVID-19 AE. The synergistic effect of the diffuse and chronic fragility of the endothelium vessels and the proinflammatory state encountered in patients with OSA would predispose patients with COVID-19 to develop a severe form of encephalopathy, with a higher risk of strong disability. It is of interest also to note that untreated OSA was reported to increase the risk of post-operative delirium, which may be related to altered vascular cerebral regulation [30].

One of the first line treatment option for OSA is Continuous Positive Airway Pressure (CPAP) therapy. A previous study demonstrated the effect of CPAP therapy on improving endothelial dysfunction related to OSA [14]. Therefore, OSA may be an actionable risk factor to be targeted to reduce chronic inflammation [31] and prevent severe forms of COVID-19 disease. Moreover, meta-analyses demonstrated that CPAP reduced the 24-h mean blood pressure, and uncontrolled studies and prospective clinical cohorts suggested that CPAP was able to reduce the number of fatal and non-fatal cardiovascular events, including arrhythmias, myocardial infarction and stroke [13, 32]. Despite a high prevalence in the specific multimorbid population with severe forms of COVID-19, OSA remains widely underdiagnosed [33]. Screening and treating OSA over the global population could have a major impact on public heath by both preventing COVID-19 severity and vascular complications.

Our study has some limitations. The mNOSAS score assesses the risk for significant OSA and not about definite OSA assessed by polysomnography which was unfeasible at the acute phase of COVID-19 AE. Therefore, information on the severity of OSA (the apnea hypopnea index) and the hypoxic burden (known to be associated with endothelial dysfunction) is missing. Among the patients with definite OSA, some had a severe form of OSA requiring the use of a nocturnal device. However, although this device could not be used in the acute phase of COVID-19, it would have been interesting to know the presymptomatic compliance of the use of this device in order to estimate its protective effect on the vascular endothelium (MRI outcome) and on the mRS at discharge. The prevalence of COVID-19 AE in our hospital is certainly underestimated, as patients hospitalized for SARS-CoV-2 infection were not systematically screened by a neurologist, but evaluated only when the referral physicians (internists or intensivists) asked for a neurological consult; patients with subtle signs may have not been identified by the referral physicians. Although we followed strict inclusion criteria for COVID-19 AE (delirium or subsyndromal delirium without cause, based on the RASS and the CAM scores), not every patient may have the opportunity to perform a comprehensive paraclinical investigation: injected MRI in 63.9%, EEG in 82.5%, and CSF analysis in 38.1% of patients. Although we could not definitively rule out encephalitis in the 60 patients without CSF analyses, the clinical decision to exclude this diagnosis was based on the follow‐up and common medical knowledge at the time of the pandemic, namely that SARS-CoV-2 rarely produces encephalitis [2]. Although circumferential enhancement of vessels is suggestive of endothelial inflammation, which is the current main hypothesis for AE, we do not bring pathological evidence in this study.

## Conclusions

This study demonstrated an association between a high risk of OSA and the development of severe COVID-19 encephalopathy and related-disability. Improved screening for OSA, which is currently underdiagnosed in the world population, and prevention of the vascular complications of OSA by promoting therapeutic devices (such as continuous positive airway pressure therapy), which are known to reverse part of endothelial dysfunction, could be a major public health issue to temper the serious consequences of COVID-19 such as encephalopathy.

### Supplementary Information

**Additional file 1: Supplemental Table 1.** Comparison of patient demographic characteristics between definite OSA group and No OSA group.

**Additional file 2: Supplemental Table 2.** Comparison of patient characteristics at the time of COVID-19 onset and COVID-19 acute encephalopathy between definite OSA group and No OSA group.

**Additional file 3: Supplemental Table 3.** Cerebrospinal fluid analyses at the time of COVID-19 acute encephalopathy.

## Data Availability

The data that support the findings of this study are available from the corresponding author upon reasonable request. Some data may not be made available due to privacy or ethical restrictions.

## Abbreviations

CAM: Confusion Assessment Method
COVID-19 AE: COVID-19 acute encephalopathy
CSF: Cerebrospinal fluid
OSA: Obstructive sleep apnea
mNOSAS: Modified NOSAS score (acronym for neck circumference, obesity, snoring, age and sex)
mRS: Modified Rankin Scale
RASS: Richmond Agitation Sedation Scale
