# Supplementary material for: Obstructive sleep apnea: a major risk factor for COVID-19 encephalopathy?
Source: BMC Neurol. 2023 Sep 27;23:340. doi: 10.1186/s12883-023-03393-2 (PMC10523731; doi:10.1186/s12883-023-03393-2)
Supplement: Supplementary file 1 — Additional file 1: Supplemental Table 1. Comparison of patient demographic characteristics between definite OSA group and No OSA group. [file 12883_2023_3393_MOESM1_ESM.docx]

**Supplemental Table 1. Comparison of patient demographic characteristics between definite OSA group and No OSA group.**

| variables | Total (n=45) | Definite OSA (n=27, 60%) | No OSA (n=18, 40%) | p-value |
| --- | --- | --- | --- | --- |
| age (at admission) | 67.9 (+/- 11.1) | 69.6 (+/- 10.9) | 65.3 (+/- 11.3) | 0.126^2^ |
| male | 35 (77.8 %) | 24 (88.9 %) | 11 (61.1 %) | 0.064¹ |
| education degree* |  |  |  | 0.906¹ |
| 1 | 7 (15.6 %) | 4 (14.8 %) | 3 (16.7 %) |  |
| 2 | 15 (33.3 %) | 10 (37.0 %) | 5 (27.8 %) |  |
| 3 | 11 (24.4 %) | 6 (22.2 %) | 5 (27.8 %) |  |
| ≥ 1 vascular risk factors | 35 (77.8 %) | 24 (88.9 %) | 11 (61.1 %) | 0.065¹ |
| Body Mass Index (BMI) | 28.2 (+/- 5.9) | 31.4 (+/- 5.4) | 23.5 (+/- 2.2) | < 0.001^2^ |
| smoking | 5 (11.1 %) | 3 (11.1 %) | 2 (11.1 %) | 0.999¹ |
| blood pressure hypertension | 30 (66.7 %) | 20 (74.1 %) | 10 (55.6 %) | 0.333¹ |
| diabete | 21 (46.7 %) | 16 (59.3 %) | 5 (27.8 %) | 0.077¹ |
| dyslipidemia | 15 (33.3 %) | 10 (37.0 %) | 5 (27.8 %) | 0.747¹ |
| apnea hypopnea index (/h)** | 53.68 (±24.92) | 53.68 (±24.92) | - | - |
| night breath device for OSA** | 13 (28.9 %) | 13 (48.1 %) | 0 (0.0 %) | - |
| modified NOSAS score** | 10.1 (+/- 4.7) | 13.4 (+/- 2.5) | 5.1 (+/- 1.8) | < 0.001^2^ |
| preexisting cognitive disorder*** | 6 (13.3 %) | 3 (11.1 %) | 3 (16.7 %) | 0.676¹ |
| preexisting heart disease*** | 11 (24.4 %) | 9 (33.3 %) | 2 (11.1 %) | 0.271¹ |
| preexisting respiratory disease*** | 6 (13.3 %) | 5 (18.5 %) | 1 (5.6 %) | 0.638¹ |
| toxic use*** | 4 (8.9 %) | 3 (11.1 %) | 1 (5.6 %) | 0.640¹ |

Supplemental Table 1 legend:

¹ Fisher's exact test. Table results were given in number of patients (percentage of total number of patients per group).

^2^ t-test. Table results were given in mean (± standard deviation).

* Education degree was defined as followed: 1=primary education, 2=lower secondary education, 3=upper secondary education.

** Definite OSA was assessed by polysomnography (gold standard).^12,15^ The Apnea Hypopnea Index was used to indicate the severity of definite OSA. The Apnea Hypopnea Index is the number of apneas or hypopneas recorded per hour of sleep (number of events per hour). Based on the Apnea Hypopnea Index, the severity of OSA is classified as follows: None/Minimal: < 5 per hour; Mild: ≥ 5, but < 15 per hour; Moderate: ≥ 15, but < 30 per hour; Severe: ≥ 30 per hour.^12,15^ Among patients with definite OSA, some of them usually used night devises such as continuous positive airway pressure (CPAP) or oral appliances like the mandibular advancement device.^12^ The NOSAS score classified patients at high risk for significant OSA with the following items: neck circumference, obesity, snoring, age and sex (NOSAS).^18^

*** Preexisting cognitive disorder was defined as any cognitive disorder impairment by a neurologist before the beginning of COVID-19 acute encephalopathy. Preexisting heart and respiratory disease were defined as any heart or respiratory disease diagnosed by a cardiologist, a pulmonologist or a general practitioner prior to the onset of COVID-19 acute encephalopathy. Toxic use was reported by the physician in charge at the time of hospital admission (anamnesis/heteroanamnesis).

Abbreviation: OSA= obstructive sleep apnea.
