# Supplementary material for: Obstructive sleep apnea: a major risk factor for COVID-19 encephalopathy?
Source: BMC Neurol. 2023 Sep 27;23:340. doi: 10.1186/s12883-023-03393-2 (PMC10523731; doi:10.1186/s12883-023-03393-2)
Supplement: Supplementary file 2 — Additional file 2: Supplemental Table 2. Comparison of patient characteristics at the time of COVID-19 onset and COVID-19 acute encephalopathy between definite OSA group and No OSA group. [file 12883_2023_3393_MOESM2_ESM.docx]

**Supplemental Table 2. Comparison of patient characteristics at the time of COVID-19 onset and COVID-19 acute encephalopathy between definite OSA group and No OSA group.**

| variables | Total (n=45) | Definite OSA (n=27, 60%) | No OSA (n=18, 40%) | p-value |
| --- | --- | --- | --- | --- |
| **General examination and parameter at COVID-19 onset** | | | | |
| dyspnea | 26 (57.8 %) | 14 (51.9 %) | 12 (66.7 %) | 0.498^1^ |
| cough | 31 (68.9 %) | 21 (77.8 %) | 10 (55.6 %) | 0.212^1^ |
| fever | 37 (82.2 %) | 24 (88.9 %) | 13 (72.2 %) | 0.235^1^ |
| FiO2 | 30.1 (+/- 14.7) | 31.6 (+/- 16.6) | 27.8 (+/- 11.4) | 0.913^2^ |
| **Neurological signs at** **COVID-19 acute encephalopathy** | | | | |
| fluctuation | 37 (82.2 %) | 23 (85.2 %) | 14 (77.8 %) | 0.694¹ |
| inattention | 35 (77.8 %) | 23 (85.2 %) | 12 (66.7 %) | 0.166¹ |
| thought disturbance | 26 (57.8 %) | 19 (70.4 %) | 7 (38.9 %) | 0.031¹ |
| alertness trouble | 17 (37.8 %) | 13 (48.1 %) | 4 (22.2 %) | 0.149¹ |
| drowsiness | 22 (48.9 %) | 13 (48.1 %) | 9 (50.0 %) | 0.999¹ |
| agitation | 14 (31.1 %) | 10 (37.0 %) | 4 (22.2 %) | 0.470¹ |
| psychomotor slowdown | 24 (53.3 %) | 16 (59.3 %) | 8 (44.4 %) | 0.417¹ |
| obnubilation | 16 (35.6 %) | 12 (44.4 %) | 4 (22.2 %) | 0.239¹ |
| perseveration | 22 (48.9 %) | 16 (59.3 %) | 6 (33.3 %) | 0.067¹ |
| disorientation | 17 (37.8 %) | 12 (44.4 %) | 5 (27.8 %) | 0.319¹ |
| hallucination | 8 (17.8 %) | 5 (18.5 %) | 3 (16.7 %) | 0.999¹ |
| focal neurological sign | 12 (26.7 %) | 8 (29.6 %) | 4 (22.2 %) | 0.735¹ |
| **COVID-19 acute encephalopathy features** | | | | |
| CAM | 2.6 (+/- 1.2) | 3.0 (+/- 1.1) | 2.1 (+/- 1.2) | 0.008^3^ |
| RASS ≤ -3 | 2 (4.4 %) | 1 (3.7 %) | 1 (5.6 %) | 0.999^1^ |
| mutism | 7 (15.6 %) | 5 (18.5 %) | 2 (11.1 %) | 0.684¹ |
| severe encephalopathy* | 27 (60.0 %) | 22 (81.5 %) | 5 (27.8 %) | < 0.001¹ |
| duration of encephalopathy (days) | 48.2 (+/- 95.5) | 66.4 (+/- 118.4) | 18.5 (+/- 11.8) | 0.356^3^ |
| **Biological results in the blood** | | | | |
| C-reactive protein (mg/l) | 64.3 (15.4-143.1) | 57.9 (24.1-121.7) | 90.1 (9.7-159.3) | 0.857^2^ |
| leucocytes (/mm3) | 9.97 (8.08-13.30) | 9.67 (7.92-12.21) | 11.35 (8.52-14.16) | 0.427^2^ |
| lymphocytes (/mm3) | 0.88 (0.60-1.16) | 0.85 (0.57-1.09) | 0.97 (0.70-1.19) | 0.522^2^ |
| segmented neutrophils (/mm3) | 7.55 (5.74-10.80) | 7.38 (5.44-10.04) | 9.09 (6.42-11.68) | 0.528^2^ |
| monocytes (/mm3) | 0.50 (0.35-0.69) | 0.42 (0.34-0.69) | 0.58 (0.39-0.68) | 0.599^2^ |
| thrombocytes (/mm3) | 261 (191-331) | 258.00 (240-334) | 264(167-319) | 0.672^2^ |
| **Brain MRI** | | | | |
| leucoencephalopathy |  |  |  | <0.001¹ |
| 0 | 6 (13.3 %) | 0 (0.0 %) | 6 (33.3 %) |  |
| 1 | 16 (35.6 %) | 14 (51.9 %) | 2 (11.1 %) |  |
| 2 | 3 (6.7 %) | 2 (7.4 %) | 1 (5.6 %) |  |
| 3 | 7 (15.6 %) | 3 (11.1 %) | 4 (22.2 %) |  |
| stroke (DWI lesion) | 13 (28.9 %) | 5 (18.5 %) | 8 (44.4 %) | 0.104¹ |
| hyperT2 lesion (number) | 8.0 (+/- 6.0) | 9.1 (+/- 5.5) | 6.3 (+/- 6.5) | 0.163^3^ |
| microbleed (number) | 2.9 (+/- 6.2) | 2.4 (+/- 6.5) | 3.6 (+/- 5.9) | 0.164^3^ |
| number of vessels with endotheliitis*** |  |  |  | < 0.001¹ |
| 0 | 8 (17.8 %) | 0 (0.0 %) | 8 (44.4 %) |  |
| 1 | 4 (8.9 %) | 2 (7.4 %) | 2 (11.1 %) |  |
| 2 | 5 (11.1 %) | 5 (18.5 %) | 0 (0.0 %) |  |
| 3 | 9 (20.0 %) | 9 (33.3 %) | 0 (0.0 %) |  |
| circumferential endotheliitis*** | 17 (37.8 %) | 16 (59.3 %) | 1 (5.6 %) | < 0.001¹ |
| endotheliitis*** |  |  |  | < 0.001¹ |
| unilateral | 8 (17.8 %) | 6 (22.2 %) | 2 (11.1 %) |  |
| bilateral | 10 (22.2 %) | 10 (37.0 %) | 0 (0.0 %) |  |
| **EEG slowing** | 19 (42.2 %) | 12 (44.4 %) | 7 (38.9 %) | 0.848¹ |
| **Epidemiological features** | | | | |
| intensive care unit | 32 (71.1 %) | 20 (74.1 %) | 12 (66.7 %) | 0.840^3^ |
| hospitalization time | 41.0 (27.0-60.5) | 40.0 (27.0-60.0) | 42.5 (30.0-62.75) | 0.730^2^ |
| mRS at discharge |  |  |  | 0.030^1^ |
| 0 | 5 (11.1 %) | 4 (14.8 %) | 1 (5.6 %) |  |
| 1 | 13 (28.9 %) | 3 (11.1 %) | 10 (55.6 %) |  |
| 2 | 8 (17.8 %) | 4 (14.8 %) | 4 (22.2 %) |  |
| 3 | 7 (15.6 %) | 6 (22.2 %) | 1 (5.6 %) |  |
| 4 | 7 (15.6 %) | 6 (22.2 %) | 1 (5.6 %) |  |
| 5 | 1 (2.2 %) | 1 (3.7 %) | 0 (0.0 %) |  |
| 6 (death) | 4 (8.9 %) | 3 (11.1 %) | 1 (5.6 %) |  |
| mRS at discharge ≥ 3 | 19 (42.2 %) | 16 (59.3 %) | 3 (16.7 %) | 0.012¹ |

Supplemental table 2 legend: This table presents patient characteristics at the time of COVID-19 onset (general examination data and COVID-19 pulmonary imaging status) and at COVID-19 acute encephalopathy paroxysm (neurological signs, encephalopathy features, epidemiological features, biological data from blood and CSF, brain MRI data and EEG results).

¹ Fisher's exact test. Table results were given in number of patients (percentage of total number of patients per group).

^2^ Mann-Whitney u test. Table results were given in median (± interquartile ratio).

^3^ t-test. Table results were given in mean (± standard deviation).

* severe encephalopathy was defined on a RASS < −3 at worst presentation ─ meaning deep sedation, no response to voice but possible movement or eye, opening to physical stimulation; or on a CAM score ≥ 3 among patients with a RASS ≥ -3 ─ meaning displaying 3 out of 4 items among symptoms fluctuation, inattention, thought disturbance, and altered alertness.

** Missing value. Brain MRI were missing in many patients because of the inability to perform these tests due to patient compliance at the acute phase of COVID-19 encephalopathy.

***The term “endotheliitis” referred to homogeneous gadolinium contrast enhancement of the inner part of the vessel wall (injected brain MRI) without stenosis. Circumferential endotheliitis referred to contrast enhancement of the vessel wall greater than 50% of the circumference.

Abbreviations: CAM = Confusion Assessment Method, COVID-19 AE = COVID-19 acute encephalopathy, DWI = diffusion-weighted imaging, OSA = obstructive sleep apnea, mRS = modified Rankin Scale, RASS = Richmond Agitation Sedation Scale.
