# Supplementary material for: Obstructive sleep apnea: a major risk factor for COVID-19 encephalopathy?
Source: BMC Neurol. 2023 Sep 27;23:340. doi: 10.1186/s12883-023-03393-2 (PMC10523731; doi:10.1186/s12883-023-03393-2)
Supplement: Supplementary file 3 — Additional file 3: Supplemental Table 3. Cerebrospinal fluid analyses at the time of COVID-19 acute encephalopathy. [file 12883_2023_3393_MOESM3_ESM.docx]

| Biological results in the CSF Total OSA No OSA | | | | |
| --- | --- | --- | --- | --- |
| *missing values* 60* | | *46* | *14* |  |
| leucocytes (/cm3) | 3.9 (±14.6) | 4.3 (±15.5) | 0.5 (±0.6) | 0,056^1^ |
| lymphocytes (/cm3) | 2.0 (±1.9) | 2.1 (±2.0) | 0.9 (±0.1) | 0.005^2^ |
| neutrophils (/cm3) | 0 (0-0) | 0.01 (0 - 0) | 0 (0 - 0) | 0,519^1^ |
| monocytes (/cm3) | 0.08 (0-0.2) | 0.08 (0 - 0.2) | 0.1 (0.1 - 0.1) | 0,757^1^ |
| macrophages (%) | 1.0 (±1.3) | 1.1 (±1.3) | 0.4 (±0.5) | 0,301^1^ |
| albumin quotient/Qlim | 1.26 (±0.94) | 1.27 (±0.96) | 1.18 (±0.88) | 0.845^1^ |

**Supplemental Table 3. Cerebrospinal fluid analyses at the time of COVID-19 acute encephalopathy.**

Supplemental table legend:

^1^ Mann-Whitney u test. Table results are given in median (± interquartile ratio).

^2^ t-test. Table results are given in mean (± standard deviation).

*Missing values. Lumbar puncture (CSF testing) are missing in many patients because of the inability to perform it due to patient compliance at the acute phase of COVID-19 encephalopathy.

Abbreviation: Qlim= albumin quotient cut‐off corrected by age.
